# Recent changes in mountain grasslands: a vegetation resampling study

**DOI:** 10.1002/ece3.1987

**Published:** 2016-03-06

**Authors:** François Gillet, Leslie Mauchamp, Pierre‐Marie Badot, Arnaud Mouly

**Affiliations:** ^1^ Université Bourgogne Franche‐Comté – CNRSUMR 6249 Chrono‐environnement16 route de Gray25030Besançon CedexFrance; ^2^ Ecole Polytechnique Fédérale de LausanneLaboratory of Ecological SystemsStation 21015LausanneSwitzerland

**Keywords:** Anthropogenic changes, community diversity, CSR strategies, diachronic survey, ecological indicator values, grassland, Jura Mountains, resampling vegetation plots, vegetation dynamics

## Abstract

Understanding how land‐use changes affect different facets of plant biodiversity in seminatural European grasslands is of particular importance for biodiversity conservation. As conclusions of previous experimental or synchronic observational studies did not converge toward a general agreement, assessing the recent trends in vegetation change in various grassland systems using a diachronic approach is needed. In this resurvey study, we investigated the recent changes in grassland vegetation of the French Jura Mountains, a region with a long tradition of pastoralism. We compared the floristic composition of 150 grassland plots recorded between 1990 and 2000 with new relevés made in 2012 on the same plots. We considered taxonomic, phylogenetic and functional diversity as well as ecological characteristics of the plant communities derived from ecological indicator values and life strategies of the species. PCA of the floristic composition revealed a significant general trend linked to the sampling year. Wilcoxon paired tests showed that contemporary communities were generally more dominated by grass species and presented a higher tolerance to defoliation, a higher pastoral value, and a higher nutrient indicator value. Comparisons revealed a decrease in phylogenetic and functional diversity. By contrast, local species richness has slightly increased. The intensity of change in species composition, measured by Hellinger distance between pairs of relevés, was dependent on neither the time lag between the two surveys, the author of the first relevé nor its location or elevation. The most important changes were observed in grasslands that previously presented low pastoral value, low grass cover, low tolerance to defoliation, and high proportion of stress‐tolerant species. This trend was likely linked to the intensification of grassland management reported in the region, with a parallel increase in mowing frequency, grazing pressure, and fertilization level. More restrictive specifications should be applied to agricultural practices to avoid overexploitation of mountain species‐rich grasslands and its negative consequences on their biodiversity and resilience.

## Introduction

Grassland ecosystems often comprise high plant species richness at fine scale and thus contribute to biodiversity conservation at larger scale. Specifically, seminatural, temperate European grasslands, managed for a long time with low‐intensity grazing or mowing, are the communities with the world records for vascular plant species richness at very small spatial grain (Wilson et al. 2012). Beyond conservation concerns, the maintenance of high biodiversity in grasslands is of first interest as it supports many ecosystem services and determines the level and the stability of these services (e.g., Tilman and Downing 1994; Tscharntke et al. 2005; Balvanera et al. 2006; Mauchamp et al. 2013). As predicted by the insurance hypothesis (Yachi and Loreau 1999), a positive effect of species richness on productivity has been found in artificial grasslands, increasing over time (Reich et al. 2012). A recent meta‐analysis of experimental studies on such artificial species assemblages suggested that species richness generally improves resistance of grassland productivity to climatic extreme events, but not its recovery rate (Isbell et al., 2015). Recent removal experiments in seminatural pastures of the Swiss Jura Mountains have shown that subordinate plant species, never dominant but mainly responsible for species richness in the plant community, play a crucial functional role by enhancing the resistance to climatic hazards and by increasing the adaptability of the whole grassland ecosystem to environmental changes (Mariotte et al. 2013). In temperate natural and seminatural grasslands, local species richness shows unimodal relationship with productivity (Dengler et al. 2014).

Recent studies recorded a dramatic decrease in plant diversity and especially species richness over the last decades in various grassland types across European regions. In Central Europe, the comparison of historical relevés with resampled ones revealed a loss of 30–50% of species at the plot level that was associated with changes in functional composition. Species‐poorer modern communities became dominated by mowing‐tolerant grass species with high nutrient requirements, while the cover of ruderal species decreased (Wesche et al. 2012). A similar study in the Swiss Alps highlighted a loss of specificity in mountain hay meadows, which revealed a recent homogenization of plant diversity at a regional level (Homburger and Hofer 2012).

These observations could be related to changes in grassland management that have occurred in the last decades (Wesche et al. 2012). Indeed, recent studies have focused on two opposite tendencies affecting grassland vegetation, both aiming at maximizing productivity (Plantureux et al. 2005; Buttler et al. 2009). On the one hand, the less productive areas that are located far away from the farm or not easily accessible for hay making and to tractors are progressively neglected and tend to be afforested. On the other hand, an intensification of the most productive parcels is observed, especially those that are located near the farm building and with an easy access for machines. Both of these trends may conduct to biodiversity loss (Tasser and Tappeiner 2002; Mottet et al. 2006; Wesche et al. 2012). However, release or abandonment of grassland management in many regions may lead to the opposite, that is, positive, effect on local vascular plant species richness, especially in semidry grasslands by an enrichment in frindge and woody species through progressive succession (e.g., Jandt et al. 2011; Vassilev et al. 2011).

Among management practices, intensification often associates an increase in defoliation intensity (mowing and grazing) and in nutrient inputs (Gaujour et al. 2012). An increase in grazing intensity leads to a decrease in species richness (Farruggia et al. 2006) and to the dominance of ruderal and competitive species at the expense of stress‐tolerant species (Gaujour et al. 2012). Under very high grazing pressure, ruderal species adapted to high levels of disturbance replace competitive species. Short grasslands under high grazing pressure tend also to be dominated by species that present a rosette architecture, early flowering, and seed dispersal to avoid livestock grazing (Garnier and Navas 2012; Gaujour et al. 2012).

Fertilization effects enhance those of defoliation as it is known that high fertilization rates increase the dominance of fast‐growing species, which have a high ability to uptake nutrients from the soil (Plantureux et al. 2005), with negative effects on species diversity (Hejcman et al. 2010). In intensive hay meadows, high industrial fertilizer inputs favor the development of ruderal species as well as high abundance of tall grasses and competitive forbs (Marini et al. 2007). The rapid growth of competitive species and their high capacity to uptake nutrients from the soil allow them to dominate in such fertile environments.

Several prominent studies of vegetation change in grasslands along the last decades considered species richness (e.g., Homburger and Hofer 2012; Wesche et al. 2012). However, this metric is considered an incomplete indicator for grassland biodiversity when used alone (especially in the response of vegetation to management, Wilsey et al. 2005), is highly dependent on the sampling effort (Gotelli and Colwell 2001), and is changing more slowly than species abundance (Scimone et al. 2007). For those reasons, the use of a variety of taxonomic, phylogenetic, and functional diversity indices is recommended for biodiversity assessment (de Bello et al. 2010; Dengler et al. 2014; Perronne et al. 2014). Some recent studies compared different metrics of biodiversity in semiarid rangelands and dry grasslands (e.g., Hanke et al. 2014; Niu et al. 2014; Reitalu et al. 2014). Depending on the geographic and ecological context, conclusions of these studies may greatly differ. To our knowledge, no resampling study comparing taxonomic, phylogenetic, and functional diversity metrics of grasslands based on a common framework has been carried out so far. Moreover, there is no agreement and poor scientific knowledge on the impact of global change on plant biodiversity for seminatural mesic productive meadows and pastures, albeit widespread in medium‐mountain European landscapes.

The aim of this study was to assess the changes affecting recently plant communities in the permanent grasslands of the French Jura Mountains, a large traditionally pastoral region devoted to dairy farming and Protected Designation of Origin (PDO) cheese production. Severe specifications are designed to ensure a strong link between the quality of the product and of its terroir. It is therefore important for PDO cheese industry to acquire scientific knowledge about grassland biodiversity, associated ecosystem services, and their response to anthropogenic changes. In this study, we compare two floristic surveys conducted at the same sites after more than 10 years to address the following questions:

1. Is there a general trend in compositional changes from the 1990s to present across this region?
2. Can we relate the observed changes in species composition to significant variations in ecological indicator values and life strategies? In particular, is there any indication for a change in fertility and defoliation regime?
3. These changes are they associated with a decrease in species richness and other taxonomic, phylogenetic, and functional metrics of alpha, beta, and gamma diversities?
4. Can we relate the intensity of compositional change observed for each pair of relevés to some characteristics of the first relevés, such as observer identity, elevation, or initial grassland composition?

## Materials and Methods

### Study area

**Figure 1:**
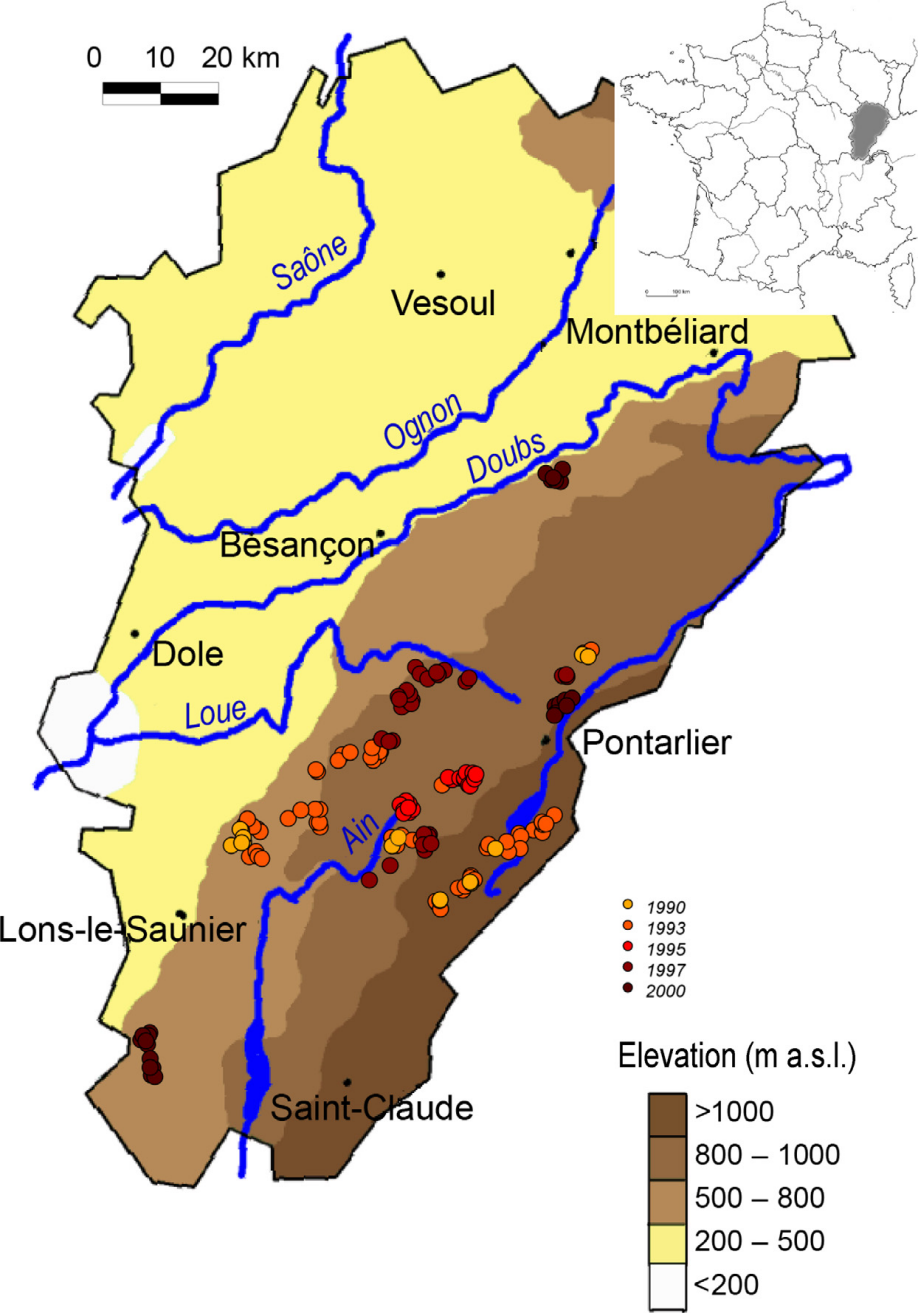
Geographical location of the 150 past floristic relevés resampled in 2012.

Our resampling study was carried out in the northwestern part of the French Jura Mountains, in the Franche‐Comté region (Fig. 1). The study area includes three main structural units across an elevation gradient: first plateau (500–800 m a.s.l.), second plateau (800–950 m a.s.l.), and high range (950–1700 m a.s.l.). Climate is nemoral with a strong suboceanic influence. Across the elevation gradient (from Orgelet to La Dôle), the mean annual precipitation ranges from about 1000–2000 mm and the mean annual temperature from 9.6 to 2.4°C (http://en.climate-data.org/). Predominant soils are cambisols developed on limestone with a variable superficial cover of silt deposed by wind. Permanent grasslands represent 22% of the area in the study region and are mainly used for dairy farming and PDO Comté cheese production. Indeed, the production of Comté cheese represents a major economic sector in the Franche‐Comté region and the most important PDO cheese industry in France. Such production implies the existence of constraining specifications, especially for agricultural practices applied to grasslands. In particular, the average nutrient inputs are capped to 120 kg N ha^−1^ y^−1^ and the stocking rate must not exceed 1.3 adult bovine units per hectare.

### Plot selection

We used a database obtained from a previous pluriannual collaborative program between Comité Interprofessionnel de Gestion du Comté (CIGC) and our laboratory. We gathered 1072 phytosociological relevés of grasslands made during the 20th century and precisely located by their GPS coordinates in the PDO Comté area. Among these relevés, we made a first selection of plots recorded between years 1990 and 2000, in order to have a time interval of at least 10 years before the new plot records. All retained plots have been projected on recent aerial orthophotographs to keep those that are nowadays located in a parcel still used as grassland (excluding situations of agricultural conversion). Of this dataset, 150 plots were finally randomly selected to conduct the second survey in spring 2012 (Fig. 1). In this sample, 50 relevés belonged to the *Arrhenatherion elatioris* W. Koch 1926 (lowland mesic hay meadows), 45 to the *Triseto flavescentis–Polygonion bistortae* Braun‐Blanquet & Tüxen ex Marschall 1947 (mountain mesic hay meadows), and 55 to the *Cynosurion cristati* Tüxen 1947 (mesic pastures) (Ferrez 2007).

### Vegetation resampling

Each plot was relocalized in the field from its original geographic coordinates (including elevation) using a GPS and a map. The location error depends on the precision of the original coordinates and may be considered less than 10 m. The vegetation was recorded in May–June 2012 at the same place and at the same month than the first relevé. We applied the same classical phytosociological approach: inside a plot of 200 m^2^ around the GPS point, all observed vascular plant species were listed and the cover of each species was estimated using the seven degrees of the Braun‐Blanquet scale (*r*, +, 1, 2, 3, 4, 5). These codes were then converted to absolute percentage cover (van der Maarel 1979) and finally to relative cover by summing to 100% for each plot.

### Diversity metrics

Taxonomic diversity was measured by species richness (N0), inverse Simpson diversity (N2), and species evenness (N20 = N2/N0), based on Rényi general entropy and species number equivalents (Hill 1973). Rao quadratic diversity with Jost correction was used to compare taxonomic, phylogenetic, and functional facets of diversity, as it corresponds to a generalization of the inverse Simpson index (Jost 2007; de Bello et al. 2010). We compared alpha diversity metrics at the plot level and also the overall spatial gamma diversities and their additive partitioning at the survey level, that is, for old versus new datasets.

The computation of phylogenetic diversity implies the construction of an ultrametric phylogenetic tree composed of the vascular plant species recorded during the field work. To do so, we used sequences of two genes encoding chloroplast proteins (rbcL and matK) and calibrated branch length following the detailed procedure given in Mauchamp et al. (2014).

The computation of functional metrics was based on a set of functional traits, chosen according to previous studies that revealed their interest for studying vegetation variation along various gradients (Pakeman 2004; Louault et al. 2005; Quétier et al. 2007; Ansquer et al. 2009; Martin et al. 2009; Garnier and Navas 2012). We considered traits related to plant morphology (Hmax: maximum vegetative height; LD: leaf distribution), leaf characteristics (LDMC: leaf dry matter content; SLA: specific leaf area), and reproductive strategies (SM: seed mass; CGO: clonal growth organs). These traits were extracted from various databases: LEDA Traitbase for SM, LDMC, and SLA (Kleyer et al. 2008); CLO‐PLA for CGO (binary variables for 11 types represented in our dataset among the 17 forms contained in the database of Klimešová and de Bello 2009); Biolflor for LD (Klotz et al. 2002); and Jäger et al. (2000) for Hmax. LD is a qualitative variable classifying species according to the distribution of leaves on the stems: (1) rosette; (2) semi‐rosette; (3) leaves distributed regularly along the stem; (4) shoot scarcely foliated; (5) tufts and crowns, leaves concentrated as a rosette at the top of taller shoot; and (6) other types (Klotz et al. 2002). In addition to these true functional traits (*sensu* Violle et al. 2007), we also considered CSR strategies (Grime et al. 2007), based on a tradeoff among resistance to stress (S), adaptation to disturbance (R), and competitive ability (C); C, S, and R numeric values represent the triangular coordinates, summing to 1 for each species. For each of these traits, we computed a separate functional diversity index based on relative cover and trait value of each plant species in the relevé.

### Functional and ecological descriptors of plant communities

The functional composition of each community was given by the usual agronomic plant life‐form classification, which distinguishes grasses, forbs, and legumes. We also considered the sociological–ecological groups of plant species (van der Maarel 1993; Mauchamp et al. 2014), that is, characteristic of specific habitats according to the regional phytosociological classification: oligotrophic grasslands (lawn), productive pastures (pasture), productive mown meadows (hayfield), oligotrophic fringes (fringe), eutrophic fallows (fallow), forest understorey (forest), and wet grasslands or fens (swamp). The relative cover of the different plant life forms and sociological–ecological groups was calculated in each sampled plot by summing the relative cover of the corresponding species. In addition, community‐weighted means of CSR strategies (Grime et al. 2007) were calculated to compare the contributions of competitive (straC), stress‐tolerant (straS), and ruderal (straR) strategies in the plant community.

We considered the ecological indicator values given by Landolt et al. (2010), as floristic composition gives useful information on ecological conditions. They include values related to climate (light ivL, temperature ivT, continentality ivK) and soil (moisture ivF, nutrient ivN, aeration ivD, humus content ivH, and pH ivR) conditions. Mean ecological indicator values were computed at the community level by weighting the species values according to their niche width, that is, giving twice more weight to species with narrow requirements (Landolt et al. 2010) without taking into account species relative cover. We considered cover‐weighted mean values for the defoliation tolerance (defol), the importance of allochthonous flora (neo, based on the type and time of immigration of the flora), and the degree of artificialization (artif, influence of man on site conditions, that is, “urbanity” *sensu* Wittig et al. 1985), based on Flora indicativa (Landolt et al. 2010). As Landolt indicator value of defoliation tolerance is mixing mowing and herbivory (grazing and browsing), we also considered separate indicators for mowing (MowTol), grazing (GraTol), and trampling (TraTol) tolerance, based on Biolflor database (Klotz et al. 2002). This set of community indicators was complemented by the pastoral value (PV), based on the agronomic forage quality of the species (Daget and Poissonet 1971).

### Statistical analyses

To extract the main gradients of vegetation composition, we performed a principal component analysis (PCA) on the Hellinger‐transformed species‐cover matrix (Borcard et al. 2011). To assess to what extent the two‐first axes of the PCA reveal a temporal trend or an elevation gradient, the numeric variables “year” and “elevation” were fitted and projected *a posteriori* on the PCA plot using the envfit() function from the vegan R package. The same method was applied to community descriptors derived from the floristic composition, in order to relate the principal components to variations in functional composition, ecological indicator values, and diversity indices.

Redundancy analysis (RDA) was performed on the Hellinger‐transformed species matrix constrained by the sampling year to measure and to test the proportion of variance explained by this single explanatory variable, considered as numeric (Borcard et al. 2011).

We then compared all 32 community descriptors (Landolt ecological indicator values, taxonomic, phylogenetic, and functional diversity indices, sociological–ecological groups of plants, plant life forms, CSR strategies) between the two surveys using paired Wilcoxon signed rank tests, with *a posteriori* Holm correction for multiple comparisons. We chose nonparametric Wilcoxon test instead of paired t‐test because 14 of 32 descriptors did not meet the assumption of normally distributed differences between paired observations (Supporting Information, Table S1).

In addition, we measured the intensity of compositional change in each plot by the Hellinger distance between the old and the new relevé. These distances were then related to three sets of explanatory variables: (i) those concerning methodological aspects that could have biased the observed changes in vegetation, namely the author of the first relevé, the elapsed time between the two relevés (Julian days), and the difference in day‐of‐the‐year; (ii) those related to the geographic location of the sites (longitude, latitude, and elevation); and (iii) those describing the plant community during the first survey (functional descriptors, diversity metrics). For this purpose, we applied a series of simple linear models with Hellinger distance as response variable and tested the significance of each explanatory variable by considering adjusted R^2^ and *P*‐values, without and with Holm correction.

Statistical analyses were performed using R 3.2.0 (http://www.R-project.org) and the packages vegan, ade4, and agricolae.

## Results

**Figure 2:**
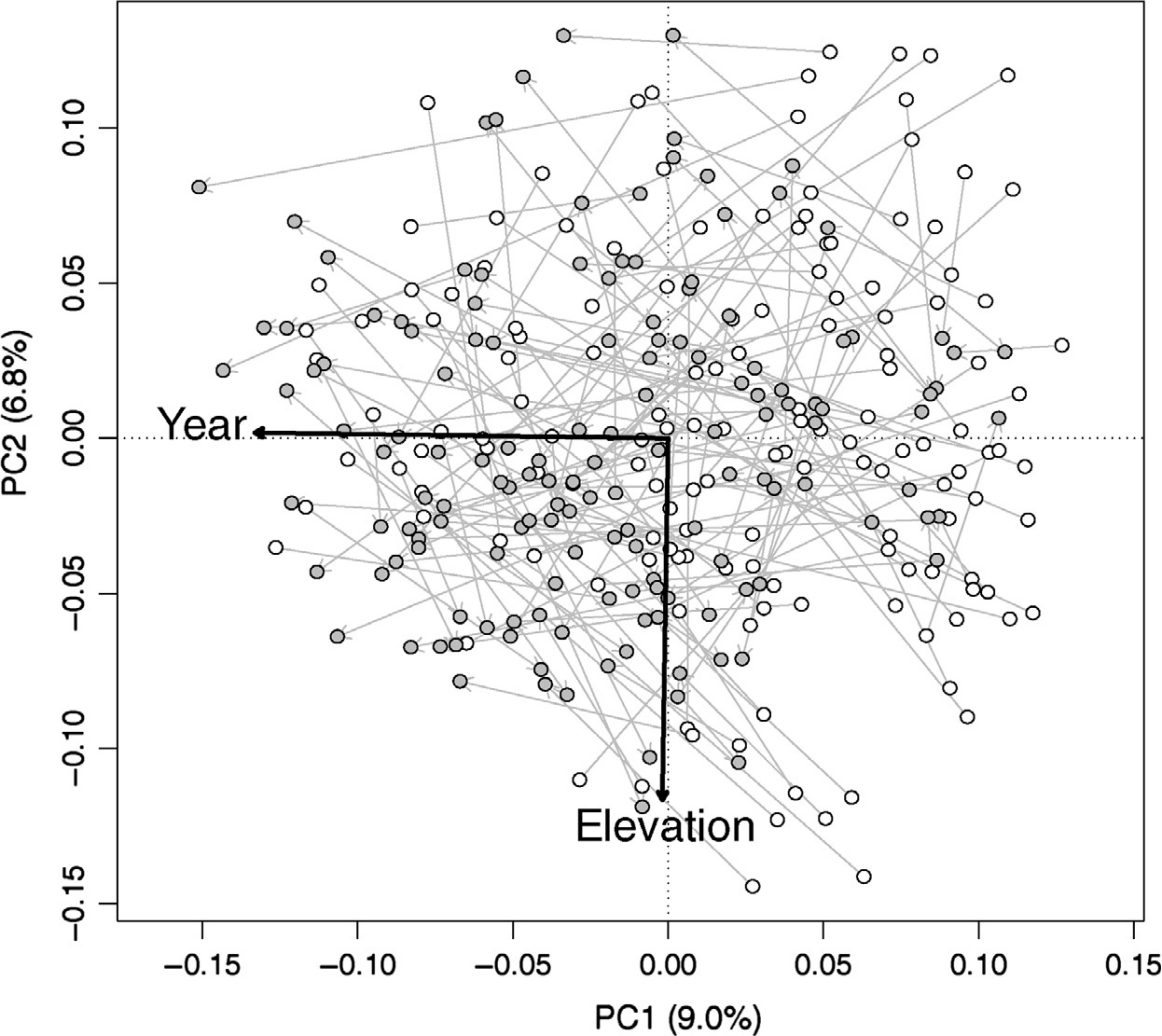
Principal component analysis of the Hellinger‐transformed species composition: sample plots. Each gray arrow represents the shift in floristic composition for each plot from the first survey (white points) to the second survey (gray points). The year and elevation of the relevés were projected *a posteriori* on the PCA plot.

**Figure 3:**
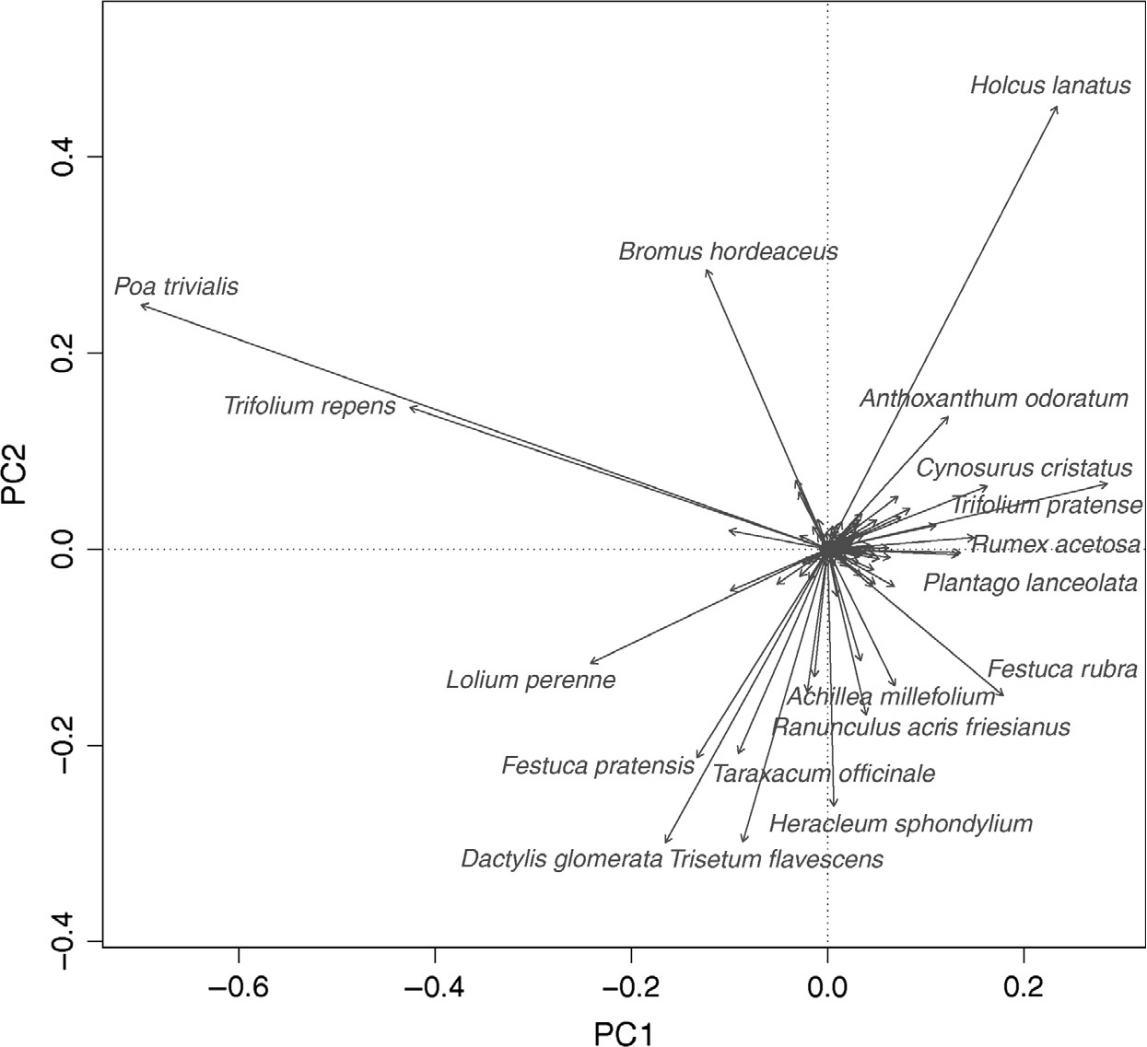
Principal component analysis of the Hellinger‐transformed species composition: species. Only species far from the origin are labeled.

The PCA projection revealed in most cases important changes in species composition between the two surveys, as shown by the length of the arrows linking old and new relevés (Fig. 2). Axes 1 and 2 represented 15.8% of the variance of the Hellinger‐transformed species matrix. The *a posteriori* projection of sampling year and elevation showed that these two variables were strongly correlated to axes 1 and 2, respectively. This indicates that the main gradient of floristic composition was markedly linked to the year of the observations and that this general trend was independent of the elevation. Being positively correlated to this temporal gradient, dominance of ubiquitous grassland species such as *Poa trivialis*,* Trifolium repens,* and *Lolium perenne* appeared to have increased over time (Fig. 3). However, according to RDA, the sampling year (considered as numeric) explained only 2.1% of the variance of the vegetation data (adjusted *R* ^2^, *P *=* *0.001 after 1000 permutations). Indeed, this weak but significant temporal trend was partly hidden by reverse trajectories in some grasslands, as shown by divergent arrows in the PCA plot (Fig. 2).

**Figure 4:**
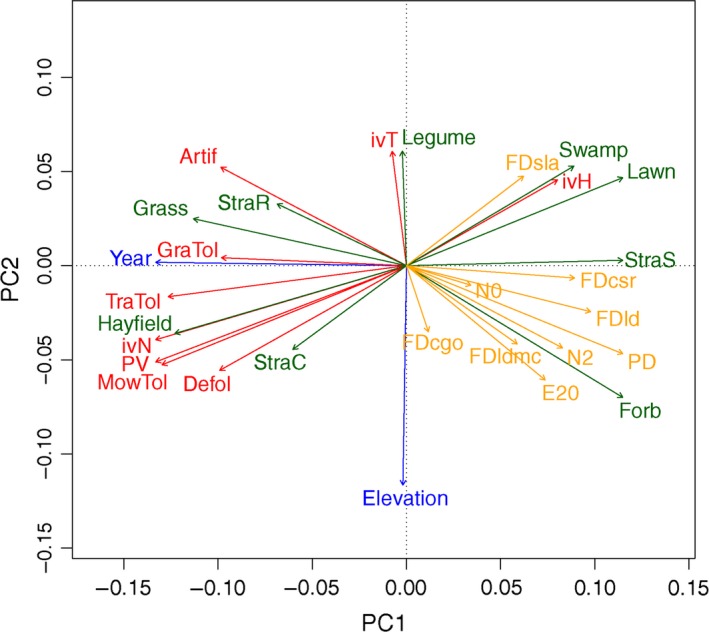
Principal component analysis of the Hellinger‐transformed species composition: fitted variables. Green arrows: functional composition (CSR strategies, sociological–ecological groups, life forms); red arrows: ecological indicator values; orange arrows: diversity indices. See Table 1 for an explanation of abbreviations.

Nevertheless, fitting community descriptors on PCA first axes helped to interpreting this temporal trend (Fig. 4). Competitive (straC) and ruderal (straR) plant species, mostly Poaceae (grass), which are frequent in productive hayfields but are tolerant to both mowing, grazing, and trampling tended to gain in importance over years at the expense of nonlegume dicots (forb), oligotrophic (lawn, swamp), and stress‐tolerant (straS) species. Ecological indicator values suggested an increase in nutrient availability (ivN), disturbance level (artif, defol, GraTol, MowTol, TraTol), and pastoral value (PV), and a decrease in soil organic matter (ivH). Most of taxonomic, phylogenetic, and functional diversity metrics were negatively correlated to this temporal gradient, indicating an overall decline of grassland biodiversity.

**Figure 5:**
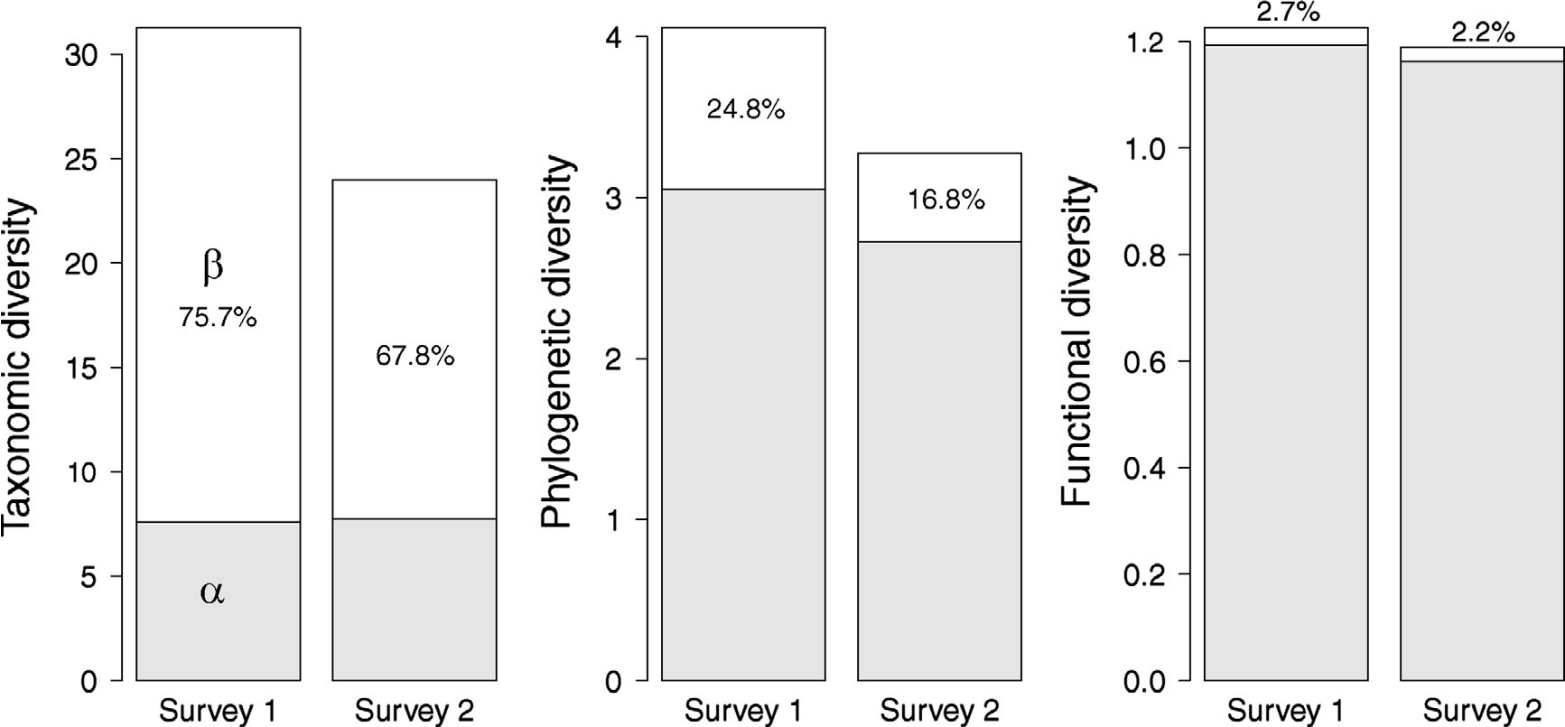
Comparison of alpha, beta, and gamma diversities between surveys 1 and 2. Gamma diversity is the sum of mean alpha diversity (gray bar) and beta additive diversity (white bar). Percentages are proportions of beta diversity in the additive partitioning of gamma diversity. Taxonomic diversity is inverse Simpson index N2.

The additive partitioning of diversity provided complementary information at the survey level (Fig. 5). Mean alpha (intrasite) taxonomic diversity increased very slightly, while beta (intersite) diversity decreased markedly, resulting in a lower regional gamma diversity. Both alpha and beta diversities decreased considering phylogenetic and functional facets. The proportion of (additive) beta phylogenetic diversity was lower than that for taxonomic metrics but higher than that for functional metrics.

According to Wilcoxon paired tests (Table 1, Supporting Information Fig. S1), the plant communities recorded in 2012 generally showed a higher tolerance to anthropogenic disturbances (defol, GraTol, MowTol, TraTol), higher nutrient requirements (ivN), lower light requirements (ivL), a higher proportion of grass species, a lower proportion of forbs, and present a higher pastoral value (PV). In addition, these pairwise comparisons showed that more recent communities presented lower phylogenetic diversity (PD) and functional diversity for CSR strategies (FDcsr), seed mass (FDsm), leaf distribution (FDld), and clonal growth organs (FDcgo). By contrast, among taxonomic diversity metrics, plant communities recorded in 2012 presented a higher species richness (N0). Other differences were not significant, at least after Holm correction of *P*‐values. In particular, there was no evidence of an increase in exotic or invasive species (neo), change in taxonomic diversity (N2) or CSR dominant strategy. Interestingly, indicator values of temperature (ivT) and pH (ivR), often related to global change, did not show significant differences.

**Table 1:** Results of the paired Wilcoxon signed rank tests comparing 32 community descriptors between old and new relevés made in each plot (*n* = 150). *P*‐values, before and after Holm adjustment for multiple comparisons, are given for two alternative hypotheses: decrease (greater for old relevés) or increase (less for old relevés). *P < 0.05; **P < 0.01; ***P < 0.001. Significant results in bold

		Alternative hypothesis: decrease		Alternative hypothesis: increase	
		*P*	*P* (Holm)	*P*	*P* (Holm)
Taxonomic and phylogenetic diversity					
N0	Species richness	0.999	1.000	0.001**	0.019*
E20	Species evenness N2/N0	0.005**	0.119	0.995	1.000
N2	Simpson diversity	0.283	1.000	0.718	1.000
PD	Rao phylogenetic diversity	<0.001***	0.004**	1.000	1.000
Functional diversity					
FDhmax	Maximum height	0.981	1.000	0.019*	0.431
FDsm	Seed mass	<0.001***	<0.001***	1.000	1.000
FDldmc	LDMC	0.006**	0.153	0.994	1.000
FDsla	SLA	0.122	1.000	0.878	1.000
FDld	Leaf distribution	0.001**	0.029*	0.999	1.000
FDcgo	Clonal growth organs	0.001**	0.018*	0.999	1.000
FDcsr	CSR strategy	<0.001***	<0.001***	1.000	1.000
CSR strategies					
straC	Competitive ability	0.981	1.000	0.019*	0.431
straS	Stress tolerance	0.119	1.000	0.881	1.000
straR	Disturbance tolerance	0.272	1.000	0.729	1.000
Ecological indicator values					
ivT	Temperature	0.775	1.000	0.226	1.000
ivK	Continentality	0.037*	0.824	0.963	1.000
ivL	Light	<0.001***	<0.001***	1.000	1.000
ivN	Soil nutrients	1.000	1.000	<0.001***	<0.001***
ivR	Soil pH	0.047*	0.988	0.953	1.000
ivD	Soil aeration	0.010**	0.241	0.990	1.000
ivF	Soil moisture	0.441	1.000	0.559	1.000
ivH	Soil organic matter	0.404	1.000	0.597	1.000
defol	Defoliation tolerance	1.000	1.000	<0.001***	<0.001***
GraTol	Grazing tolerance	1.000	1.000	<0.001***	<0.001***
MowTol	Mowing tolerance	1.000	1.000	<0.001***	<0.001***
TraTol	Trampling tolerance	1.000	1.000	<0.001***	<0.001***
Other indices					
neo	Importance of allochthonous flora	0.801	1.000	0.200	1.000
artif	Artificialization degree	0.994	1.000	0.006**	0.148
PV	Pastoral value	1.000	1.000	<0.001***	<0.001***
grass	Relative cover of grasses	1.000	1.000	<0.001***	<0.001***
forb	Relative cover of forbs	<0.001***	<0.001***	1.000	1.000
legume	Relative cover of legumes	0.055	1.000	0.945	1.000

Linear models showed that the intensity of change in floristic composition between old and new relevés (Table 2) was not explained by methodological constraints, namely the identity of the observer (author), the year of the first relevé (nominal year), the elapsed time between the two surveys (delayd in days), or the difference in day‐of‐the‐year sampling period (delaydy). Moreover, Hellinger distance between first and new relevés neither depended on geographic coordinates nor elevation of the site. But it was explained by some characteristics of the plant community at the first observation. Stress‐tolerant communities with high taxonomic, phylogenetic, and functional diversity (computed for CSR) underwent more changes. Conversely, nutrient‐rich, artificialized, intensively mown or grazed grasslands with high pastoral value were less affected.

**Table 2:** Results of simple linear regression models explaining the intensity of change in each plot by various potential sources of variation: methodological constraints, geographical location, or characteristics of the plant community at the first survey (*n* = 150). The table gives *P*‐values before and after Holm correction, the adjusted coefficient of determination and the sign of the slope of the corresponding linear model if significant. *P < 0.05; **P < 0.01; ***P < 0.001. Significant results in bold

		*P*	*P* (Holm)	Adj. *R*²	Sign of slope (%)
Methodological constraints					
Author	Identity of the first observer	0.141	0.985	2.0	
Year	Year of the first observation (nominal)	0.392	1.000	0.1	
delayd	Time elapsed between the 2 observations (days)	0.496	1.000	0.0	
delaydy	Difference in day‐of‐the‐year (days)	0.524	1.000	0.0	
Geographical location					
Longitude	Longitude E (decimal degrees, GWS84)	0.910	1.000	0.0	
Latitude	Latitude N (decimal degrees, GWS84)	0.731	1.000	0.0	
Elevation	Elevation (m a.s.l.)	0.664	1.000	0.0	
Diversity indices of the old community					
N2	Taxonomic diversity (inverse Simpson)	0.019*	0.152	3.0	+
PD	Phylogenetic diversity (Rao)	0.005**	0.047*	4.5	+
FDcsr	Functional diversity (CSR)	<0.001***	<0.001***	12.8	+
Indicator values of the old community					
straS	Stress tolerance	<0.001***	0.001**	10.2	+
ivN	Nutrient availability	<0.001***	0.005**	7.4	−
defol	Defoliation tolerance	<0.001***	0.003**	7.9	−
artif	Artificialization degree	<0.001***	0.001**	9.1	−
PV	Pastoral value	<0.001***	<0.001***	11.9	−

## Discussion

### Changes in community composition

This diachronic study revealed significant changes in the plant species composition of permanent grasslands in the French Jura Mountains, linked to the ecological and functional attributes of the communities. Paired tests revealed that more recent plant communities indicated more defoliation and nutrient supply. These results are consistent with a previous study, which compared the plant community attributes between the 1950 and 1960s and the 21st century in Central European grasslands (Wesche et al. 2012). Using Ellenberg ecological indicator values, the authors highlighted an increase of mowing‐tolerant and nitrogen‐demanding competitive grasses at the expense of stress‐tolerant species. They mentioned that the changes they recorded were mainly due to local nutrient inputs, rather than regional climate change. This increase in nutrient availability over time was attributed to agricultural management and nitrogen atmospheric depositions. van den Berg et al. (2011) also revealed that calcareous grasslands of the United Kingdom, studied over a 10‐year period, presented changes in species composition due to nitrogen deposition, with a decrease in the characteristic species of calcareous grasslands.

The proportions of plant life forms could also present variations, as shown by McGovern et al. (2011) in *Agrostis–Festuca* uplands of the United Kingdom. After 40 years, the plant communities underwent an increase in the grass:forb ratio. In accordance with these findings, our results showed an increase in the relative cover of grasses associated with an increase in the pastoral value, indicating a higher forage quality regarding productivity, nutritive value, and digestibility for cattle (Al Haj Khaled et al. 2006; Farruggia et al. 2008).

### Changes in diversity metrics

Our comparison of various functional diversity metrics revealed significant differences between the two sets of relevés. The plant communities underwent a decrease in functional diversity for CSR strategies and several functional traits, which was likely explained by a loss of the extreme trait values.

Functional diversity is linked to the coexistence of species that respond differently to disturbance (Chapin et al. 1997; Yachi and Loreau 1999; Laliberté et al. 2010). This allows maintaining ecosystem stability and resilience (Elmqvist et al. 2003). In our study area, the loss of functional diversity that occurred in the past decades could increase ecosystem vulnerability to future disturbances, such as climatic events (Laliberté et al. 2010).

Our diachronic study also revealed a loss of phylogenetic diversity since the 1990s. Stability and resilience of communities are linked to the diversity of species and the niches they occupy. Evolutionary history represented by co‐occurring species has been shown to be an important predictor of ecosystem function and phylogenetic distances surrogate ecological differences (Cadotte et al. 2012). Indeed, phylogenetic diversity summarizes the trait space within a community, comprising complex physiological or biochemical traits that are not easily quantifiable but may be important for the understanding of community assembly. In our study, phylogenetic and functional diversity metrics showed parallel decreasing trends for all traits except maximum height. Moreover, we observed a significant negative relationship between phylogenetic diversity and indicators of land‐use intensification. This suggests that the stability of the studied grasslands became lower, as well as the ability to respond to environmental variations, due to the shrinkage of the trait space in the plant communities. It also illustrates the phylogenetic conservatism in land‐use sensitive traits for the French Jura grassland plant species. Interestingly, by studying the effect of intensification of agricultural practices on the evolution of German grassland plant communities, Egorov et al. (2014) found no phylogenetic signal related to intensification but a significant decrease in species richness.

Despite these important changes in phylogenetic and functional diversity between the two surveys, we did not record an overall decrease in species richness and Simpson diversity in the studied grasslands. We even observed a slightly higher number of species in recent relevés. These results contrast with several other studies that reported a decrease in species richness in the last decades (Gaudnik et al. 2011; Homburger and Hofer 2012; Wesche et al. 2012; Roth et al. 2013). Roth et al. (2013) showed that species richness for vascular plants was negatively correlated with nitrogen depositions in the Swiss Alpine and Jura mountain grasslands; the decrease in species richness has been linked to the decline of oligotrophic species outcompeted by eutrophic species. van den Berg et al. (2011) also reported a decrease in species richness, Shannon diversity and evenness in calcareous grasslands among the ten past years in UK, in relation to nitrogen deposition rates. However, focusing on dry calcareous grasslands in Germany, Diekmann et al. (2014) reported no decline in species richness along the last 70 years despite the loss of specialists of these habitats, attributed to cumulated nitrogen deposition.

The contrasted results obtained for species richness compared to literature might be partly due to differences in sampling effort among observers between the two surveys. Indeed, species richness is known to be very sensitive to the sampling effort (Gotelli and Colwell 2001). However, this authorship bias is certainly low, as all observers (6 in the first survey, 3 in the second survey) applied a similar protocol. Moreover, our tests have shown that the identity of the first observer had no influence on the measured intensity of change in species composition. A more plausible explanation is the time lag, as we considered a period of 12–22 years between the first investigations and our new relevés. Indeed, most of the studies that registered a decrease in species richness considered a longer time lag between the two surveys (e.g., Wesche et al. 2012: 50 years; Homburger and Hofer 2012: 60 years). A recent long‐term experiment revealed, for example, that it took more than 14 years to observe changes in species richness in fertilized plots, while aboveground biomass showed a rapid response to this fertilization increase (Dickson and Gross 2013). As vegetation response, especially qualitative changes in species composition, may require several decades (Scimone et al. 2007), the time interval we considered could be not long enough to observe a decrease in species richness. Further observations in the same plots would be necessary to address this question.

### Determinants of the intensity of change

The intensity of vegetation change in each plot, measured by Hellinger distance between old and new relevés, was influenced neither by methodological constraints, nor by the geographical location and elevation of the site (Table 2). The independence of the magnitude of change from elevation is an interesting unexpected result, given the common evidence of slower vegetation dynamics at higher elevation (Tasser and Tappeiner 2002). This finding can be connected to results of our indirect gradient analysis, showing that the temporal trend is independent of the elevation (temperature) gradient (Fig. 2). However, this analysis also showed that changes in ecological and functional characteristics of the plant communities were slightly different along the elevation gradient (Fig. 4). For example, increased artificialization associated with ruderal strategy appeared to prevail at low elevation, whereas increased mowing intensity and nutrient availability associated with competitive species tended to prevail at higher elevation.

Moreover, the intensity of change was dependent on various ecological and functional characteristics of the plant communities of the 1990s. The most important changes in floristic composition occurred in formerly stress‐tolerant, extensively used grasslands with low productivity but high phylogenetic and functional diversity (Table 2). Conversely, formerly highly productive grasslands with high defoliation and fertilization rates have undergone less vegetation change. This result is explained by the susceptibility of lower productive pastures and hayfields to land‐use intensification. A long‐term experiment conducted in Germany showed that oligotrophic, poorly productive grasslands may be progressively replaced by productive grasslands after various fertilization treatments (Hejcman et al. 2007; Chytrý et al. 2009).

### Causes and consequences of observed vegetation changes

Recent changes in species and functional composition of grasslands recorded in many studies have been linked to increased nutrient inputs (from agricultural management and/or atmospheric depositions) that are even considered as the major threat on plant species richness (Dickson et al. 2014). Increasing fertilization rates favor the dominance of competitive and ruderal species at the expense of stress‐tolerant ones (Marini et al. 2007). The response of vegetation to increased fertilization regimes may be delayed because of the rear effects due (i) to the application of organic fertilizers that implies a first mineralization by soil organisms before nutrient become available for plant uptake (Kirkham et al. 2008; Gaujour et al. 2012), but also (ii) to the high persistence of phosphorous in the soil (Parfitt et al. 2005; Ceulemans et al. 2014).

Thus, the most likely explanation for the general trend we observed from our data is a recent intensification of agronomic practices in permanent grasslands in the study region, with an increase in both fertilization and defoliation intensities during the past two decades. This is confirmed by personal communications with farmers in the French Jura Mountains, who recognize that increased performances of dairy cows combined with new farm organization schemes led to a new vision of maximized production in the 1990s, associated with more pressure on grasslands to ensure and secure a high level of milk production (Michaud 2003).

Notwithstanding, the existence of strict specifications in the PDO Comté area may still have limited these changes. However, it is now clear that they have not been sufficient to prevent the erosion of functional and phylogenetic diversity in permanent grasslands, even in mountain regions once thought to be spared.

Consequences on the environment and on ecosystem services could be detrimental to the sustainability of the PDO cheese industry if this trend continues in the future. In particular, the functioning of grassland ecosystems, for example, their ability to store carbon, to prevent nutrient loss, to resist to climatic events, and to adapt to global warming, relies on their biodiversity. At the farm and regional levels, it should be paid attention to preserve beta diversity, by maintaining beside highly productive hayfields a sufficient area of extensively and diversely used, species‐rich grasslands, which have undergone the most important damages in the last decades.

## Conflict of Interest

None declared.

## Supporting information


**Fig. S1.** Comparison of eight community descriptors between surveys 1 and 2. **Table S1.** Descriptive statistics of 32 community descriptors computed from original and resampled floristic relevés of 150 grassland plots in the French Jura Mountains.
