# Supplementary material for: Recent changes in mountain grasslands: a vegetation resampling study
Source: Ecol Evol. 2016 Mar 6;6(8):2333–45. doi: 10.1002/ece3.1987 (PMC4782259; doi:10.1002/ece3.1987)
Supplement: Supplementary file 1 — Fig. S1. Comparison of eight community descriptors between surveys 1 and 2. Table S1. Descriptive statistics of 32 community descriptors computed from original and resampled floristic relevés of 150 grassland plots in the French Jura Mountains. [file ECE3-6-2333-s001.docx]

**Supporting Information**

**Table S1** Descriptive statistics of 32 community descriptors computed from original (Survey 1) and resampled (Survey 2) floristic relevés of 150 grassland plots in the French Jura Mountains. Results of paired t-tests are given for descriptors showing normally distributed differences between paired observations (Shapiro test, ‘Yes’ if *P* > 0.1): ns not significant; ms *P* < 0.1, * *P* < 0.05, ** *P* < 0.01, *** *P* < 0.001. See Table 1 for an explanation of abbreviations.

|  | Survey 1 | | |  | Survey 2 | | |  | Shapiro-test | | Paired t-test | |
| --- | --- | --- | --- | --- | --- | --- | --- | --- | --- | --- | --- | --- |
|  | median | mean | sd |  | median | mean | sd |  | *P* |  | *P* |  |
| N0 | 27.000 | 27.747 | 8.313 |  | 29.000 | 30.600 | 8.427 |  | 0.396 | Yes | 0.002 | ** |
| E20 | 0.333 | 0.364 | 0.163 |  | 0.306 | 0.307 | 0.098 |  | < 0.001 | No |  |  |
| N2 | 9.063 | 9.935 | 5.652 |  | 8.878 | 9.234 | 3.504 |  | < 0.001 | No |  |  |
| PD | 3.253 | 3.253 | 0.744 |  | 3.012 | 2.909 | 0.678 |  | 0.239 | Yes | < 0.001 | *** |
| FDhmax | 1.00829 | 1.00858 | 0.00560 |  | 1.00878 | 1.00878 | 0.00167 |  | < 0.001 | No |  |  |
| FDsm | 1.00043 | 1.00052 | 0.00032 |  | 1.00035 | 1.00040 | 0.00021 |  | < 0.001 | No |  |  |
| FDldmc | 1.212 | 1.212 | 0.058 |  | 1.194 | 1.195 | 0.058 |  | 0.291 | Yes | 0.012 | * |
| FDsla | 1.119 | 1.123 | 0.034 |  | 1.117 | 1.117 | 0.027 |  | < 0.001 | No |  |  |
| FDld | 1.396 | 1.468 | 0.341 |  | 1.260 | 1.348 | 0.288 |  | 0.506 | Yes | 0.001 | *** |
| FDcgo | 1.195 | 1.190 | 0.039 |  | 1.178 | 1.177 | 0.040 |  | 0.558 | Yes | 0.002 | ** |
| FDcsr | 1.181 | 1.196 | 0.067 |  | 1.156 | 1.165 | 0.053 |  | 0.476 | Yes | < 0.001 | *** |
| straC | 0.359 | 0.362 | 0.046 |  | 0.371 | 0.375 | 0.048 |  | 0.310 | Yes | 0.015 | * |
| straS | 0.252 | 0.253 | 0.070 |  | 0.237 | 0.241 | 0.055 |  | < 0.001 | No |  |  |
| straR | 0.390 | 0.386 | 0.064 |  | 0.388 | 0.384 | 0.057 |  | 0.103 | Yes | 0.810 | ns |
| viT | 3.380 | 3.368 | 0.095 |  | 3.360 | 3.372 | 0.091 |  | 0.225 | Yes | 0.529 | ns |
| viK | 2.980 | 2.995 | 0.096 |  | 2.970 | 2.981 | 0.090 |  | 0.431 | Yes | 0.119 | ns |
| viL | 3.760 | 3.749 | 0.087 |  | 3.710 | 3.716 | 0.088 |  | 0.003 | No |  |  |
| viN | 3.435 | 3.429 | 0.229 |  | 3.540 | 3.543 | 0.210 |  | 0.433 | Yes | < 0.001 | *** |
| viR | 3.155 | 3.167 | 0.107 |  | 3.150 | 3.153 | 0.090 |  | 0.770 | Yes | 0.093 | ms |
| viD | 2.275 | 2.224 | 0.229 |  | 2.220 | 2.182 | 0.228 |  | < 0.001 | No |  |  |
| viF | 2.930 | 2.941 | 0.191 |  | 2.930 | 2.939 | 0.145 |  | < 0.001 | No |  |  |
| viH | 3.030 | 3.084 | 0.154 |  | 3.040 | 3.068 | 0.125 |  | < 0.001 | No |  |  |
| defol | 3.660 | 3.607 | 0.306 |  | 3.810 | 3.779 | 0.207 |  | 0.217 | Yes | < 0.001 | *** |
| GraTol | 5.188 | 5.159 | 0.624 |  | 5.560 | 5.550 | 0.640 |  | 0.838 | Yes | < 0.001 | *** |
| MowTol | 6.835 | 6.746 | 0.623 |  | 7.127 | 7.068 | 0.476 |  | 0.055 | No |  |  |
| TraTol | 5.152 | 5.102 | 0.751 |  | 5.643 | 5.655 | 0.679 |  | 0.914 | Yes | < 0.001 | *** |
| neo | 1.110 | 1.205 | 0.251 |  | 1.130 | 1.228 | 0.266 |  | 0.016 | No |  |  |
| artif | 3.030 | 3.030 | 0.222 |  | 3.080 | 3.083 | 0.182 |  | < 0.001 | No |  |  |
| PV | 42.700 | 44.175 | 16.313 |  | 55.100 | 53.273 | 13.571 |  | 0.491 | Yes | < 0.001 | *** |
| grass | 0.409 | 0.420 | 0.183 |  | 0.544 | 0.551 | 0.159 |  | 0.207 | Yes | < 0.001 | *** |
| forb | 0.381 | 0.400 | 0.195 |  | 0.282 | 0.297 | 0.139 |  | 0.442 | Yes | < 0.001 | *** |
| legume | 0.158 | 0.177 | 0.130 |  | 0.143 | 0.152 | 0.100 |  | < 0.001 | No |  |  |


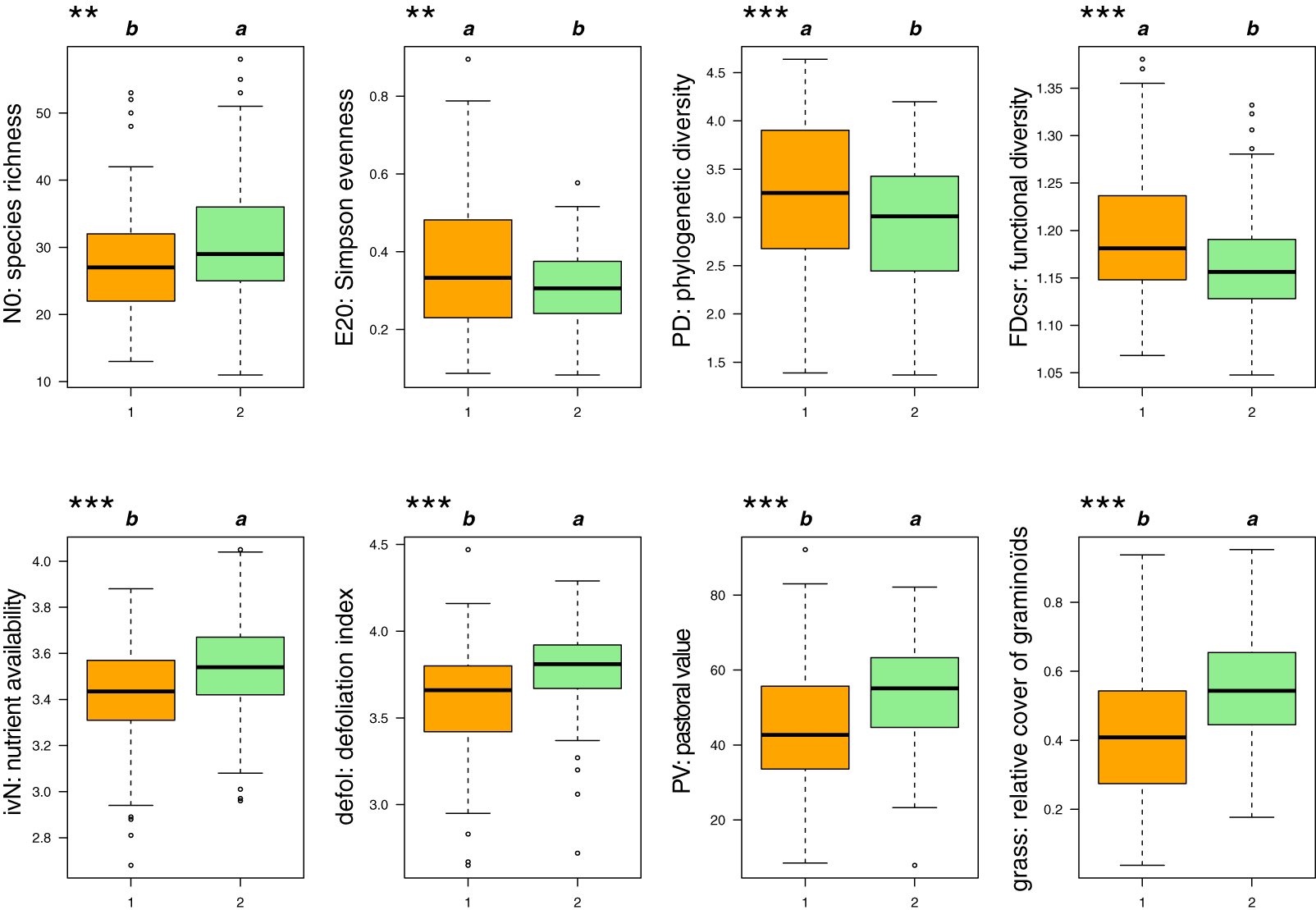


**Fig. S1** Comparison of 8 community descriptors between surveys 1 and 2. Stars and letter symbols represent results of paired, two-sided, Wilcoxon signed-rank tests: ‘a’ denotes significant higher values; ** *P* < 0.01, *** *P* < 0.001.
